# Modified ultrasound scalpel haemorrhoidectomy versus conventional haemorrhoidectomy for mixed haemorrhoids: a study protocol for a single-blind randomised controlled trial

**DOI:** 10.1186/s13063-023-07175-6

**Published:** 2023-02-24

**Authors:** Keqiang Yu, Haijun Li, Ping Xue, Zhidi Xie, Minghui Tang, Hongbo He, Jing Wu

**Affiliations:** 1 Department of Integrated Traditional Chinese and Western Medicine, West China Hospital, Sichuan University, Chengdu, Sichuan China; 2 Cheng Du Shang Jin Nan Fu Hospital, West China Hospital, Sichuan University, Chengdu, Sichuan China; 3 The Second People’s Hospital of Yibin City, Yibin, Sichuan China

**Keywords:** Modified ultrasonic scalpel haemorrhoidectomy, Milligan-Morgan, Mixed haemorrhoid, Clinical efficacy, Safety

## Abstract

**Background:**

Haemorrhoids are common and frequently occurring diseases in the clinical setting, and severe haemorrhoids require surgical treatment. There are various surgical methods to treat haemorrhoids, but each has advantages and disadvantages. In recent years, ultrasonic scalpels have been used in haemorrhoid surgery and have achieved good results. Ultrasonic scalpel haemorrhoidectomy is safer and more effective in the surgical treatment of grade III and IV haemorrhoids, with less intraoperative bleeding, less postoperative pain, and fewer complications than diathermic therapy, electrosurgical haemorrhoidectomy, PROXIMATE® PPH haemorrhoidal circular stapler haemorrhoidopexy (PPH), and traditional haemorrhoidectomy. In previous reports, the majority of ultrasonic scalpel haemorrhoidectomies were performed as open procedures, with only the body of the haemorrhoid removed with the ultrasonic scalpel and the wound left open for drainage and natural healing. However, we performed a preliminary experiment with 12 patients who underwent open ultrasonic scalpel haemorrhoidectomy in the early stage. The results showed that 8 patients had different degrees of postoperative bleeding, and 4 of them required a second haemostatic surgery under anaesthesia. Therefore, we modified the open ultrasonic scalpel haemorrhoidectomy procedure by removing the mucosa of the internal haemorrhoid and closing the base of the incision with figure-eight penetrating sutures and designed this study protocol to evaluate its clinical efficacy and safety.

**Methods:**

A randomised single-blind parallel-controlled trial is proposed for this project, and patients who meet the inclusion criteria will be divided into a test group and a control group, with 39 patients in each group. The experimental group will be treated with modified ultrasonic scalpel haemorrhoidectomy, and the control group will be treated with the Milligan-Morgan operation. The effectiveness of modified ultrasonic scalpel haemorrhoidectomy for haemorrhoids will be objectively evaluated, including the incision healing time and the time for patients to return to normal activities, postoperative complications, evaluations of anal function 3 months and 6 months after surgery, an evaluation of quality of life 6 months after surgery, and an evaluation of the patient satisfaction rate 6 months after surgery. The safety assessment will consider all adverse and serious adverse events associated with the study treatment.

**Discussion:**

The study was approved by the ethics committee. The first patient was registered on July 1 2021. The purpose of this trial will be to evaluate the clinical efficacy and safety of the modified ultrasonic scalpel haemorrhoidectomy procedure for the treatment of mixed haemorrhoids and to provide an evidence base for the clinical promotion and application of the procedure. A limitation of this study is that only the patients will be single-blinded because the researchers and the patients cannot be blinded at the same time, which may produce certain bias in the results. In addition, the sample size of this study will be small, and the test results will only represent the findings from this clinical trial. In later stages, the sample size needs to be further expanded to improve the level of evidence. Despite its limitations, we hope the present study will help provide a more optimised surgical approach in the selection of haemorrhoid surgery.

**Trial registration:**

Chinese Clinical Trial Registry (Registration ID: ChiCTR2100047229). Registered on June 11, 2021.

## Introduction

### Background and rationale

Haemorrhoids are a common and frequently occurring clinical disease, and the prevalence rate accounts for approximately 40% of the total population. The disease may occur in both men and women and may progressively worsen with age. Nonsurgical treatment does not significantly improve the symptoms of severe haemorrhoids, and they usually have irreversible pathological anatomy and physiological functions; therefore, it is necessary to treat severe haemorrhoids by surgery. At present, the commonly used surgical methods include the Milligan-Morgan operation [1], the PROXIMATE® PPH haemorrhoidal circular stapler haemorrhoidopexy (PPH) [2], and automatic haemorrhoid ligation (RPH) [3]. The Milligan-Morgan operation is a classic operation for the treatment of haemorrhoids; although it can significantly improve some clinical symptoms of patients, this procedure causes greater tissue damage and more bleeding during the operation, and the incidence of complications such as wound edge oedema and pain is higher [3]. Both PPH and RPH have some postoperative complications, such as postoperative haemorrhage and reoperation [4].

With the rapid development of minimally invasive medicine and the continuous improvement of medical instruments, there are an increasing number of studies regarding energy device procedures for haemorrhoidectomy in addition to conventional surgical devices, such as LigaSure™ haemorrhoidectomy [5, 6], ultrasonic scalpel haemorrhoidectomy, laser haemorrhoidectomy [7], and Starion haemorrhoidectomy [8]. As a new surgical equipment in clinical surgery, ultrasonic scalpels were used in clinical surgery in 1992 and are now widely used in various surgical operations. Their working principle is to make the metal cutter head oscillate at a frequency of 55.5 kHz through an ultrasonic frequency generator so that the water molecules in the tissue are vaporised, the hydrogen bonds of proteins are broken, and cells can disintegrate to cut the tissue and coagulate the blood vessels. Ultrasonic scalpels convert electrical energy into mechanical energy without electrical conduction and have high safety. Compared with traditional electric scalpels, ultrasonic scalpels cause the least damage to tissues (causing thermal damage to an area 1–3 mm wide), have less smoke, and do not cause neuromuscular stimulation [9, 10]. In recent years, ultrasonic scalpels have been gradually used in haemorrhoid operations and have shown good results. Some scholars have compared ultrasonic scalpel haemorrhoidectomy with diathermy therapy [11]. Ferguson’s with electrosurgical haemorrhoidectomy (FEH) [12], PPH [13], and traditional haemorrhoidectomy [14, 15]. The results showed that ultrasonic scalpel haemorrhoidectomy was safer and more effective in the surgical treatment of grade III and IV haemorrhoids, with less intraoperative bleeding, less postoperative pain, and fewer complications. Tsunoda A [16] studied transurethral Doppler-guided arterial mucopexy for haemorrhoids removal and ultrasonic scalptor haemorrhoidectomy for third-degree haemorrhoids. The medium-term results demonstrated that US has a faster return to normal activities. Domestic scholars Yuan Xiaoqian et al. [17] applied ultrasonic scalpel haemorrhoidectomy combined with band ligation therapy to treat mixed haemorrhoids, and the results showed that the combination was safe and effective in the treatment of mixed haemorrhoids, with the advantages of a short operation time, less intraoperative bleeding, less postoperative pain, and fast wound healing. Shen Jianyong et al. [18] used a nonligating ultrasound scalpel to treat 57 cases of circular mixed haemorrhoids. The clinical effect was obvious and there were few complications.

### Rationale for study

An ultrasonic scalpel can directly remove haemorrhoids, coagulate blood vessels and tissues, and avoid the disadvantages of massive bleeding during the ligation shedding period and postoperative recurrence after traditional surgery. However, in the preliminary experiment, we found that when only an ultrasonic scalpel was used to remove haemorrhoids without suturing the incision after the operation, the increase in intra-abdominal pressure and pressure in the anal canal due to normal defaecation activities would increase the tissue tension in the anal area. Under repeated stimulation from stool, the solidified tissues would easily crack and bleed, causing incision infection and secondary healing. Therefore, based on the previous literature and clinical studies, we have designed a modified ultrasonic scalpel haemorrhoidectomy procedure and will evaluate its clinical efficacy and safety, aiming to provide evidence for the promotion and application of the procedure in the clinical setting.

### Objectives

A randomised controlled blind study is planned to objectively evaluate the efficacy of modified ultrasonic scalpel haemorrhoidectomy in the treatment of haemorrhoids. The healing time of the incision, the time taken for patients to return to normal activities, postoperative complications, evaluations of anal function 3 months and half a year after surgery, an evaluation of the quality of life at half a year after surgery, and an evaluation of the patients’ satisfaction rates half a year after surgery will be measured.

To assess safety, the incidence of adverse events (AEs) and serious adverse events (SAEs) between the two study groups will be compared to show the potential risks for patients.

### Design

#### Setting

This will be a randomised, single-blind, parallel controlled study. Patients who meet the inclusion criteria will be divided into a treatment group and a control group, each with 39 patients. The treatment group will undergo modified ultrasonic scalpel haemorrhoidectomy, and the control group will undergo a Milligan-Morgan operation. The study will be conducted at Shangjin Hospital of West China Hospital of Sichuan University.

#### Participants

Eligible participants who meet the diagnostic criteria for haemorrhoids in the Clinical Diagnosis and Treatment Guidelines for Hemorrhoids (2006 edition), formulated by the Colorectal and Anal Surgery Group of the Chinese Medical Association Surgery Branch and the Anorectal Branch of the Chinese Society of Traditional Chinese Medicine, will be enrolled. All the participants will have been treated and hospitalised by the Department of Integrated Traditional Chinese and Western Medicine of West China Hospital of Sichuan University.

The trial has been approved by the Ethics Committee on Biomedical Research at West China Hospital of Sichuan University (Approval code: NO. 2020–367). It is also registered in the Chinese Clinical Trial Registry (Registration ID: ChiCTR2100047229).

Inclusion criteria.

1. Patients with the clinical manifestation of mixed haemorrhoids with symptoms of internal haemorrhoids and external haemorrhoids existing at the same time. Among them, internal haemorrhoids should manifest as stage III (grade) or higher;
2. Patients aged 20–70 years without obvious surgical contraindications;
3. Patients who provide informed consent, with good compliance, and are able to cooperate to complete all clinical research content.

Exclusion criteria.

1. Patients with haemorrhoids combined with anusitis;
2. Patients with haemorrhoids combined with inflammatory bowel disease;
3. Patients with suppurative infection around the anorectal canal;
4. Patients with intestinal infectious diseases (e.g. dysentery);
5. Patients diagnosed with outlet obstructive constipation.

Withdrawal criteria.

1. Patients who exhibit poor compliance during the study, which can affect the effectiveness evaluation;
2. Patients who have serious adverse events, complications and special physiological changes and are not able to continue the experiment;
3. Patients who quit on their own volition during the study;
4. Patients who withdraw from the trial, are lost to follow-up, or die due to various other reasons;
5. Patients who have incomplete data that affect the validity judgement.

#### Additional consent provisions for the collection and use of participant data and biological specimens

On the consent form, participants will be asked if they agree to use of their data should they choose to withdraw from the trial. Participants will also be asked for permission for the research team to share relevant data with people from the Universities taking part in the research or from regulatory authorities, where relevant.

This trial does not involve collecting biological specimens for storage.

#### Recruitment and trial timeline

Researchers will recruit participants from the inpatient wards of the Department of Integrated Traditional Chinese and Western Medicine. After screening patients who meet the inclusion and exclusion criteria, the researcher will inform the participants of the different treatment methods available for their condition, provide information about the study, and have the participants sign a written informed consent form. A copy will be kept by the participant, and the original will be kept at the hospital. Considering the admission to haemorrhoid surgery in our hospital and the experience of the last 2 years, it is feasible to recruit the calculated patient sample size. Other strategies to increase recruitment include announcing the study on our hospital website and providing information in the waiting room of the hospital.

**Fig. 1:**
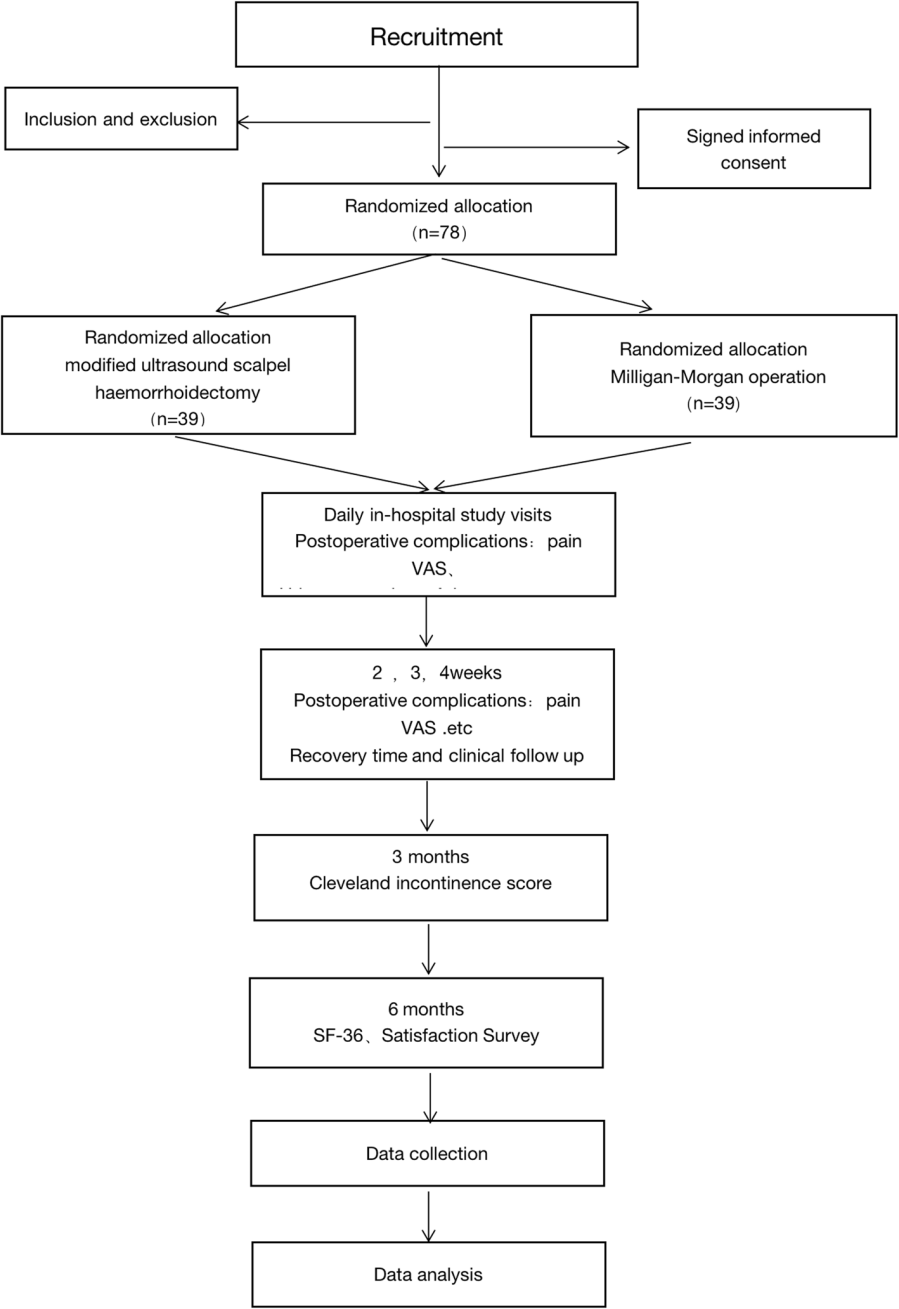
Flow chart of the research phases. VAS, visual analogue scale; SF-36, 36-Item Short Form Health Survey

The participants who meet the criteria will be registered, their baseline data and clinical features will be collected before the operation, and measurements will be taken at each observation time node according to the CRF form after the operation (Table 1). Participants who met the withdrawal criteria shall strictly follow the criteria and provide records. The registration process, the intervention and evaluation schedule, and the study flow chart are shown in Table 1 and Fig. 1.

**Table 1:** Schedule of the registration process, the intervention, and evaluations

Measures	Preoperative	Daily in-hospital study visits								Follow-up					
		POD 0	POD 1	POD 2	POD 3	POD 4	POD 5		POD ≥ 6	W 2		W 3	W 4	M 3	M 6
Incision healing time										X	X		X		
Recovery time										X	X		X		
Pain VAS		X	X	X	X	X	X	X		X	X		X		
Urinary retention		X	X	X	X	X	X	X							
Postoperative bleeding		X	X	X	X	X	X	X		X	X		X		
Postoperative oedema		X	X	X	X	X	X	X		X	X		X		
Anal stenosis	X	X	X	X	X	X	X	X		X	X		X	X	X
Cleveland incontinence score	X	X												X	X
SF-36	X	X													X
Satisfaction survey		X													X
Adverese events		X	X	X	X	X	X	X		X	X		X	X	X

*POD* Postoperative day, *W* Week, *M* Month, *VAS* Visual analogue score, *SF36* 36-Item short form health survey

#### Sample size

This study is a randomised controlled trial to evaluate modified ultrasound scalpel haemorrhoidectomy versus the Milligan-Morgan operation for mixed haemorrhoids. According to previous studies [19], the mean value of the experimental group is 10.50, the mean value of the control group is 13.40, and the standard deviation is 3.46 and 3.66, respectively. Two-sided tests is needed, with a test level of 0.05. Using the PASS 15.0 software, the sample size of the two groups is 33, and according to the 15% loss of follow-up, 78 subjects will be expected to be finally collected.

#### Randomisation and blinding

Participants will be randomly assigned equally to the two study groups with the use of simple randomisation, in which a third-party statistician will use computer software to generate pseudorandom numbers. Only the clinical data manager will have access to the random number table in the computer. Subjects will be enrolled by a research assistant in the order of enrolment time. Allocation will be made by the clinical data manager using the random number table. The statistician will be blinded to the allocation of subjects during the statistical analysis.

Since the surgical operator and the observer must know which group each subject has been assigned to, they cannot be blinded. The study has a single-blind design in which the investigators will be aware of the surgical procedures used in the different trial groups, but the participants will be unaware of them.

#### Procedure for unblinding if needed

Participants with serious adverse events will be reported to the principal investigator and the Ethics Committee (IEC) of West China Hospital, Sichuan University, who will make the decision to unblind and withdraw them from the study.

### Intervention description

#### The treatment group

1. The external haemorrhoids in the mother haemorrhoid area at points 3, 7, and 11 will be removed by an ultrasonic scalpel in a V-shape;
2. The internal haemorrhoids will be clamped with forceps to be removed, the superior haemorrhoid artery will be ligated with No. 0 mousse thread “8”, 1 ml of Xiaozhilin will be injected under the ligation proximal sticking mould, and then the haemorrhoid will be removed slowly with an ultrasonic scalpel along the clamp mark at the base;
3. The carbonisation (fixation) range of the incision will be checked, the free carbonised tissue will be removed at the incision, a needle will be inserted at the edge of the solidified tissue to suture the incision with the figure of “8” slightly above the tooth line, and then the incision epithelium will be sutured near the tooth line with the modified “8” suture method. When there is no obvious bleeding from the wound, the operation will be considered complete.

#### The control group

1. The top of the external haemorrhoids will be clamped and gently pulled outwards to expose the internal haemorrhoids. A V-shaped incision will be made on the external haemorrhoids, and the subcutaneous tissue will be stripped to 0.2 cm on the tooth line. The stripped external haemorrhoids and the base of the internal haemorrhoids will be clamped with middle-curved forceps. No. 0 silk thread will be used to run through the base root of the suture clamp. Part of the internal haemorrhoid tissue will be removed without letting the suture slip off.
2. In the same way, the other haemorrhoids will be removed and ligated, and 3 ~ 55 ml of 1:1 Xiaozhiling (1 part Xiaozhiling stock solution, 1 part normal saline) will be injected into the mucosa of the superior haemorrhoid artery area where the haemorrhoids are ligated under an anoscope to prevent bleeding. When there is no obvious bleeding from the wound, the operation will be considered complete.

### Relevant concomitant care permitted or prohibited during the trial

#### Perioperative management

After the operation, a fluid diet will be given for 1 day, and appropriate fluids will be supplemented. Nonsteroidal anti-inflammatory drugs or opioid central analgesics will be given for postoperative pain according to the VAS pain score. For postoperative urinary retention, catheterisation will be determined by the ward doctor on duty after evaluating the patient’s symptoms and signs. The patient’s catheter will be removed on POD 2. For postoperative defecation, lactulose oral solution will be used to soften the stool on the first day after the operation. If the patient still has difficulty defecating, a glycerine enema will be administered to help the patient defecate. If the symptoms still cannot be alleviated, warm normal saline can be given as a cleansing enema.

Twenty-four hours after the operation, the anal canal dressing will be removed, and the patient can defecate on their own. After defecation, the patients will be treated with heat-clearing and detoxifying traditional Chinese medicine sit-baths, iodophor disinfection, indomethacin and furazolidone embolisation of the anus, and dressing changes every day after defecation. The patient’s wound recovery, stool smoothness, and blood in the stool will be evaluated in the decision to arrange for discharge. After discharge, the patient will be advised not to exercise vigorously, continue perianal disinfection and dressing changes, and participate in regular outpatient follow-up.

#### Discharge and follow-up

Wound healing and surgical complications will be observed 1, 2, 3, and 4 weeks after the operation.

Each participant will be followed up 12 and 24 weeks after the operation via WeChat, telephone, video, and outpatient clinics. At 3 months and 6 months after the operation, the Cleveland Clinic Florida Fecal Incontinence Score (CCF-FI) will be used to evaluate the anal condition of the patients and to evaluate whether the patients have postoperative anal incontinence. The simple SF-36 quality-of-life questionnaire (homemade) will be used to evaluate the patients half a year after the operation.

For the schedule of the trial, please see Table 1.

#### Criteria for discontinuing or modifying allocated interventions

Criteria will not be applied because it is not possible for the study to change the allocation once the ultrasonic scalpel device is used during surgery. Patients will discontinue their participation in the study only if they experience a serious adverse event that may be related to the device.

#### Strategies to improve adherence to interventions

Our strategy to improve follow-up monitoring compliance includes face-to-face adherence reminders by the surgeon at regular postoperative follow-up visits. In addition, we will ask patients during the visit how best to contact them (e.g. phone, WeChat, text message). The method of reminding patients to attend follow-up visits to the questionnaire will depend on their preferred method of contact.

#### Provisions for post-trial care

There will be no provision and no compensation for patients who suffer harm as a result of their participation in the trial.

### Outcome measures

#### Primary outcome measures

The primary outcome measures are postoperative incision healing time and the time to return to normal activities, calculated in days (d) [20, 25]. The healing criteria are as follows: complete epithelialisation of the anal canal and perianal skin. The rectal mucosa should be intact, without ulcer or erosion.

Efficacy evaluation: Efficacy will be evaluated according to the efficacy evaluation for mixed haemorrhoids in the 2017 Standard for Diagnosis and Efficacy of TCM Diseases and Syndromes as follows: cured: the symptoms and haemorrhoids disappear; improved: the symptoms improve and the haemorrhoids shrink; and unhealed: there are no changes in symptoms and signs.

#### Secondary outcome measures

Postoperative complications: pain: A visual analogue scale (VAS) will be used in the two groups to observe the patients’ pain 4 h, 8 h, and 24 h after the operation and the pain from defecation for 7 consecutive days after the operation. Urinary retention will be assessed by the “with” and “without” method. Postoperative bleeding will be assessed as follows: 0 points: no bleeding; 1 point: blood on the paper; 2 points: drops of blood; 3 points: ejection of blood; and 4 points: massive bleeding that requires surgery to stop the bleeding. Postoperative oedema will be assessed as follows: 0 points: no oedema; 1 point: oedema in 1/4 of the anal margin incision; 2 points: oedema in 1/2 of the anal margin incision; 3 points: oedema in 3/4 of the anal margin incision; and 4 points: oedema around the perianal incision. Anal stenosis will be assessed by the “with” and “without” method.

Anal function evaluations 3 months and 6 months after operation: Anal function evaluation will be assessed by the CCF-FI as follows: (1) normal continence: the anus controls stool, and the passage of intestinal fluid and intestinal gas is normal; (2) partial anal incontinence: the anus cannot control intestinal fluid, intestinal gas, or loose stools; there is soiling of the underwear; or the anus has a sense of dampness; and (3) complete incontinence: the anus cannot control the passage of stool.

Evaluation of quality of life half a year after the operation: The simple SF-36 quality-of-life questionnaire (homemade) will be used to evaluate quality of life.

Evaluation of patient satisfaction rate half a year after the operation: The self-designed patient satisfaction questionnaire will be used, which is divided into very satisfied, satisfied, general, and no opinion, and calculates the percentages of the 4 levels.

#### Safety and reporting of serious adverse events

In this study, adverse events will be defined as any treatment-related medical event, including any adverse and unexpected signs, symptoms, or illnesses associated with the treatment. Adverse events will be evaluated according to the Standard for General Terminology for Adverse Events (V4.03) [21]. Within 24 h after the occurrence of adverse events, the researchers and relevant experts will evaluate and classify the events and address them in a timely manner according to the condition. We will collect, evaluate, and report any spontaneously described adverse events that occur among the participants. Adverse event data regarding the severity of the occurrence, duration (signs and symptoms) of the adverse reactions, and how to resolve them (or not) during treatment will be recorded. If the participants experience serious adverse events, they will be reported to the principal investigator and the Ethics Committee of West China Hospital of Sichuan University (IEC), and it will be decided whether the blinding should be removed and whether the participants should withdraw from the study.

### Study organisation

#### Data collection and management

The case report form (CRF) includes observation time points, outcome measurements, adverse events, and safety assessments. The evaluators of the results will follow the requirements of the CRF and fill in relevant information in a timely and accurate manner. Data collection and entry will be carried out independently by two staff members and completed by a third staff member. The principal investigator will not be involved in data collection. Data will be obtained from the CRF, and only trial group members will be able to access the CRF and perform dual data entry. The organisation of the test is as follows. The steering committee will fully oversee the design of the study. The Independent Data and Safety Monitoring Committee (IDSMC) will supervise and confirm that the CRF is completed correctly and that the data are consistent with the original data. If there are any errors or omissions, the investigator will immediately correct them. We do not intend to collect personal information about potential or registered participants other than that typically collected during hospitalisation. For confidentiality, the electronic health information will be encrypted in accordance with the hospital’s protocol. After the trial, personally identifiable information will be omitted and placed in a separate database for data analysis.

#### Composition of the coordinating centre and trial steering committee

The trial steering committee consists of three members who will meet every 3 months and review the trial. Dr. Hongbo He, as the principal investigator, will be responsible for the overall management of the trial. Dr. Jing Wu will be the clinical lead for the trial. Dr. Tang Minghui is an expert at ensuring quality control of surgery.

#### Composition of the data monitoring committee, its role and reporting structure

The Independent Data and Safety Monitoring Committee (IDSMC), consisting of two clinical experts and a statistician, will oversee the safety and quality of the data. This committee is independent and has no competing interests with the research. The IDSMC will consider protocol compliance, trial withdrawal, and safety monitoring and will make recommendations for trial continuation.

#### Statistical analysis

This study is a randomised, single-blind, parallel controlled study. The primary endpoints are the time to incision healing and the time to return to normal activities after modified ultrasonic scalpel haemorrhoidectomy. It can be considered that the recovery time after the treatment of modified ultrasonic scalpel haemorrhoidectomy is approximately normally distributed when testing the superiority hypothesis of modified ultrasonic scalpel haemorrhoidectomy and conventional haemorrhoidectomy. The hypothesis of superiority will be tested in a one-tailed approach, with a *P* value of 0.025 or less considered to indicate statistical significance.

All analyses will be performed with R 4.3.2.

#### Primary efficacy assessments

The primary efficacy measures in this trial are the time to postoperative wound healing and the time to return to normal activity. An equivalence test will be used, and the test hypothesis is as follows:

- H0: the recovery time of the intervention group-the recovery time of the control group is ≤ 0;
- Hα: the recovery time of the intervention group-the recovery time of the control group is > 0.

The recovery time of the two groups will be calculated, and the 95% confidence interval (CI) of the difference between the two groups will be determined. The difference from baseline between the trial group and the control group can be approximated as following a normal distribution, and thus the efficacy analysis will be analysed with the use of T. The superiority hypothesis will be tested between the modified ultrasonic scalpel haemorrhoidectomy group and the conventional haemorrhoidectomy group, and the test *α* = 0.025 (one-sided test). If the *p* value is less than or equal to 0.025, the superiority of postoperative recovery time of the modified ultrasonic scalpel haemorrhoidectomy group compared with the conventional haemorrhoidectomy group will be verified.

#### Secondary efficacy assessments

The statistical description and inference of the secondary indicators will be based on the characteristics of the data, and the appropriate descriptive indicators and hypothesis testing methods will be selected.

The comparison of general conditions between the two groups will be analysed by appropriate methods according to the type of indicators. The comparison between the groups of measurement data will be compared by the *t* test (homogeneity of variance, normal distribution) or the Wilcoxon rank sum test according to the distribution of data, and the chi-square test or exact probability method (if the chi-square test is not applicable) will be used for count data. The Wilcoxon rank sum test or stratified chi-square test (CMH test) will used for ranked data. Data analyses will be performed by statisticians who are independent of the study team and who are blinded to the group assignments. The acceptable significance level for all analyses will be *P* < 0.05.

#### Methods in analysis to handle protocol non-adherence and any statistical methods to handle missing data

Data will be analysed on the basis of the full analysis set (FAS), per-protocol set, and safety set. FAS analysis is a fundamental principle of intention-to-treat and will include all patients enrolled in this trial. Analyses will be performed according to the intention-to-treat principle, with patients assessed according to the group to which they were randomly assigned, regardless of actual use or algorithm adherence. The per-protocol analysis is a subset of the FAS analysis, which will include only patients who fully complete the trial. Safety set analysis refers to the set of patients who will be treated and evaluated for safety. Multiple imputation will be used to address missing values based on appropriate exploration of missing mechanisms and in accordance with good practice.

#### Plans to give access to the full protocol, participant-level data, and statistical code

The datasets analysed during the current study and statistical code are available from the corresponding author on reasonable request, as is the full protocol.

### Oversight and monitoring

#### Audits

The ethics committee will locally monitor the quality of the trial after the first batch of patients is registered. The trial management team will meet every 3 months to check the implementation of the study, including the recruitment rates, data quality, and adverse event reporting.

#### Plans for communicating important protocol amendments to relevant parties

The protocol, statistical analysis plan, data safety management plan, informed consent, and recruitment materials were reviewed and approved by the Biomedical Research Ethics Committee of West China Hospital of Sichuan University. Any subsequent modifications will be submitted for review, and annual safety and progress reports will be submitted. In addition, the online trial registration form will be updated accordingly.

#### Availability of data and materials

The final trial data for this protocol can be supplied on request.

#### Communication plan

The research results will be disseminated through peer-reviewed publications and speeches at industry academic conferences.

## Discussion

There are many surgical methods for haemorrhoids, but each has its own advantages and disadvantages. Therefore, it is particularly necessary to explore a surgical method that not only does not damage anal sphincter function but can also restore the normal shape of the anus.

The choice of surgical instruments for mixed haemorrhoids should not only take into account the different symptoms and morphological characteristics of patients with mixed haemorrhoid lesions but also local related haemodynamic changes, microcirculation control disorders, and haemorheological pathophysiological changes; the concept of “minimally invasive” should be used as much as possible to achieve a good therapeutic effect and protect the fine functions of the anus, such as defecation control. The metal head of the ultrasonic scalpel is sharp and conducive to fine anatomy and is of great value in the operation of mixed haemorrhoids. Ultrasonic scalpel haemorrhoidectomy was reported to be applied in the clinic by Jane [22] and other investigators in 2001, and research has shown that ultrasonic scalpel haemorrhoidectomy can reduce the pain and postoperative bleeding of patients. Mushaya [23] et al. conducted a meta-analysis of the comparison between ultrasonic haemorrhoidectomy and traditional haemorrhoidectomy, and the results showed that the ultrasonic scalpel group had lower pain scores, a lower complication rate, and a faster recovery than the traditional surgery group. Research by Ravi Kumar [24] et al. showed that compared with the Milligan-Morgan operation, ultrasonic haemorrhoidectomy resulted in less blood loss (19.4 and 6.1 ml, respectively). Compared with the Milligan-Morgan method group, the ultrasonic scalpel group had lower VAS pain scores on the first day and the first and the second week after the operation. There were more complications, such as postoperative bleeding and urinary retention, in the Milligan-Morgan group. In a case study of 25 patients (ultrasound scalpel group) conducted by Tariq Ahmed Mala [25], the average operation time was 17.68 ± 2.84 min, and the average operation time of the control group was 28.44 ± 3.69 min. The average blood loss of the ultrasonic scalpel group and the control group was 8.96 ± 2.15 ml and 31.72 ± 3.28 ml, respectively. VAS pain scores on the first day after surgery were 5.92 ± 0.70 in the ultrasonic scalpel group and 8.52 ± 0.84 in the control group. The ultrasound scalpel group had a lower analgesic dose, engaged in walking earlier and returned to normal work faster. In previous research reports, the vast majority of ultrasonic scalpel haemorrhoidectomy procedures were open operations only using an ultrasonic scalpel to remove the haemorrhoids; the wound was left open and drained and healed naturally. Only two studies mentioned that the incision was closed [22, 26]. Another study showed that closing the incision is not beneficial; it is not conducive to drainage of the incision and may actually slow the healing process [27]. Before the start of this study, we conducted a preliminary study: 12 patients with mixed haemorrhoids were treated with open ultrasonic scalpel resection. Unfortunately, 8 of the 12 patients had blood in the stool 5–16 days after the operation, mainly the dripping of blood and jet-like bleeding, and the amount of bleeding was ≥ 5 ml each time. Among them, 4 patients had postoperative bleeding > 50 ml. All 4 patients underwent a second haemostatic operation under anaesthesia. During the operation, the wounds were found to have ulcers and erosions and extensive bleeding after ultrasonic scalpel resection. Upon inquiry into their medical history, the 8 patients with bleeding were found to have no primary underlying diseases (hypertension, heart disease, blood system diseases, etc.) and no history of long-term anticoagulant drug use. The reasons may be as follows: if only the ultrasonic scalpel is used to remove the haemorrhoids without suturing the incision, normal defecation activity after the operation will increase the abdominal pressure and anal pressure, which will increase the tissue tension in the anal area. Under repeated stimulation from stool, the coagulated tissues will easily crack and bleed, causing incision infection and delayed healing. Therefore, we modified the open ultrasonic scalpel haemorrhoidectomy procedure. While removing the internal haemorrhoid mucosa, we sutured the base of the incision with a figure of eight and designed this research plan to evaluate its clinical efficacy and safety.

A limitation of this study is that only the patients will be single-blinded; the researchers and patients cannot be blinded at the same time, which may impose a certain bias on the results. In addition, the sample size of this study will be small, and the findings will only represent the results of this clinical trial. The sample size needs to be further expanded in later stages to improve the level of evidence. Despite its limitations, we hope this trial will help provide a more optimised surgical approach in the selection of haemorrhoid surgery.

## Trial status

Participants are still being recruited. Enrolment began in July 2021, and the trial is expected to be completed by July 2023. Approximately two thirds of the recruitment is currently completed.

## Abbreviations

PPH: PROXIMATE® PPH haemorrhoidal circular stapler haemorrhoidopexy
RPH: Automatic haemorrhoid ligation
FEH: Ferguson’s with electrosurgical haemorrhoidectomy
AEs: Adverse events
SAEs: Serious adverse events
VAS: Visual analogue score
SF-36: 36-Item Short Form Health Survey
CCF-FI: Cleveland Clinic Florida Fecal Incontinence Score
CRF: Case report form
